# Abdominal wall endometriosis (a narrative review)

**DOI:** 10.7150/ijms.38679

**Published:** 2020-02-10

**Authors:** Mara Carsote, Dana Cristiana Terzea, Ana Valea, Ancuta-Augustina Gheorghisan-Galateanu

**Affiliations:** 1 Department of Endocrinology, “Carol Davila” University of Medicine and Pharmacy, 050474, Bucharest, Romania; “C.I. Parhon” National Institute of Endocrinology, 011863, Bucharest, Romania; 2 Department of Pathology, “C.I. Parhon” National Institute of Endocrinology, 011863, Bucharest, Romania; 3 Department of Endocrinology, “Iuliu Hatieganu” University of Medicine and Pharmacy, 400012, Cluj-Napoca, Romania; 4 Department of Cellular and Molecular Biology and Histology, “Carol Davila” University of Medicine and Pharmacy, 050474, Bucharest, Romania; “C.I. Parhon” National Institute of Endocrinology, 011863, Bucharest, Romania

**Keywords:** endometriosis, abdominal wall endometriosis, caesarean scar endometriosis

## Abstract

One of the rarest forms of endometriosis is abdominal wall endometriosis (AWE), which includes caesarean scar endometriosis. AWE remains a challenging condition because some issues related to this topic are still under debate. The increasing number of caesarean sections and laparotomies will expect to increase the rate of AWE. The current incidence in obstetrical and gynaecological procedures is still unknown. The disease is probably underestimated. The pathogenic mechanism involves local environment at the implant site including local inflammation and metalloproteinases activation due to local growth factors, estrogen stimulation through estrogen receptors and potential epigenetic changes. However, the underlying mechanisms are not fully explained, and we need more experimental models to understand them. The clinical presentation is heterogeneous; the patient may be seen by a gynaecologist, an endocrinologist, a general surgeon, an imaging specialist, or even an oncologist. No particular constellation of clinical risk factors has been identified, and the histological report is the major diagnostic tool for confirmation. Surgery is the first line of therapy. Further on we need protocols for multidisciplinary investigations and approaches.

## 1. Introduction

Endometriosis, a classic topic of gynaecological endocrinology and a condition that is challenging, is characterized by the presence of endometrial epithelial and stromal cells in non-uterine locations. Endometriosis is typically associated with chronic pain and infertility and affects one in ten women of reproductive age, with different frequencies depending on the site of endometriotic implant 1. For instance, the most common pelvic locations of endometriotic tissue are the ovary and pelvic peritoneum. Sites of extra-pelvic localization include the gastrointestinal tract, the urinary tract, and the respiratory system 2,3,4. Among these, one of the rarest forms of endometriosis is abdominal wall endometriosis (AWE) or parietal endometriosis, including caesarean scar endometriosis (CSE). Post-surgical subgroups of endometriosis have increased due to the higher use of caesarean sections worldwide. To date, this particular type of implant is only partially understood, and the diagnosis is often missed and delayed 5,6,7. The effects of oestrogen exposure after caesarean section and concomitant endometrial seeding during the surgery are enhanced by chronic inflammation, altered immunity, and local growth factors 1,5,6. No particular constellation of clinical risk factors has been identified, and the histological report is the major tool for confirmation, since the preoperative diagnostic rate is low 7,8.

## 2. Materials and Methods

This is a narrative review of the literature based on research using the keywords “endometriosis”, “abdominal wall endometriosis”, and “caesarean scar endometriosis”. We mainly included articles published between 2014 and 2019. Due to the rarity of the condition, the highest level of clinical evidence from included papers are observational studies, case series, one case-control study, one prospective cohort study, and some molecular biology-based experimental studies. The aim of this article is to provide an update on AWE from a multimodal and multidisciplinary perspective.

## 3. Prevalence

AWE follows a variety of obstetrical and gynaecological surgeries that are mostly represented by caesarean sections (approximately 85% by some authors) but also comprise hysterotomy, hysterectomy, and laparoscopic procedures that are performed for non-surgical endometriosis 3,9,10. Sumathy *et al*. reported concurrent endometriosis in 18.9% of cases, while others reported no synchronous pelvic lesions 10,11. The mean age at diagnosis is 35 years, and the time from surgery to endometriosis recognition varies from 3 months to 2 decades 11,12.

The reported incidence of CSE is 0.03-0.45%; however, many authors suggest that this low number is due to the rarity of the condition and that the current incidence of AWE (including CSE) cannot be accurately evaluated since consistent epidemiological data are non-existent 13,14. Subcutaneous endometriosis near caesarean scars has been described in only a few isolated cases, including a case of cutaneous endometrial cancer 15. Recently, a case of scar endometriosis at the level of the uterine cavity was reported 16. Additionally, 18 cases of trocar port site endometriosis has been reported in the literature 17.

## 4. Pathogenic context

Even though AWE is described by some as the "iatrogenic" subtype of endometriosis, the clear explanation for why some people develop this condition after caesarean section is unclear. In addition to the technical details and precautions themselves, it seems that the pathogenic mechanism is more complex, and endocrine, immune and inflammatory pathways have been considered. While the mechanism is still an enigma, some mechanisms such as metaplasia and cell migration in association with direct seeding have been proposed 18. Intra-operative implantation is certainly not relevant to non-surgical endometriosis (or “endogenous” endometriosis), and retrograde menstruation (or the Sampson hypothesis) is not involved in post-caesarean section endometriosis, in contrast to pelvic endometriosis 19,20,21. Only a few studies have identified pre-existent pelvic endometriosis 10,11.

The local environment that allows the growth of endometrial cells and stroma includes oestrogen exposure and chronic inflammation 6,19. Angiogenic growth factor anomalies may be associated with this condition 22.

Genetic and epigenetic changes in endometrial cells are also observed in endometriosis. Genome-wide association studies have identified 12 single nucleotide polymorphisms at 10 independent genetic loci that are associated with endometriosis. Two chromosomal areas of significant linkage were observed on 10q26 and 7p13‐15 (harbouring genes such as CYP2C19, INHBA, SFRP4 and HOXA10). The identified epigenetic changes comprise methylation and demethylation of DNA and modifications of the histone code 23,24. The genetic/epigenetic theory might explain the heterogeneity of this disease with a hereditary profile, but further studies are needed.

Recently, high expression of PPAR-γ, a nuclear receptor with anti-inflammatory and neuroprotective roles, has been shown in post-operative lesions, and it has been suggested that PPAR-γ could be a pathogenic mechanism of associated pain 25. In a study focused on “iatrogenic” or “incisional” endometriosis, Lac *et al*. found that one in four women with this condition had a somatic cancer mutation that may involve two signal transduction pathways, MAPK/RAS or PI3K-Akt-mTor 20.

Non-uterine endometrial cells require metalloproteinases for local remodeling and interaction. These enzymes are activated by local factors, such as TGFβ. Itoh H *et al.* showed that stromal endometrial cells of AWE have an abnormal response to TGFβ1. This may be prevented by progesterone, which does not allow the implant to attach to the local matrix, but it seems that in AWE, there is resistance to progesterone action 26.

**Figure 1:**
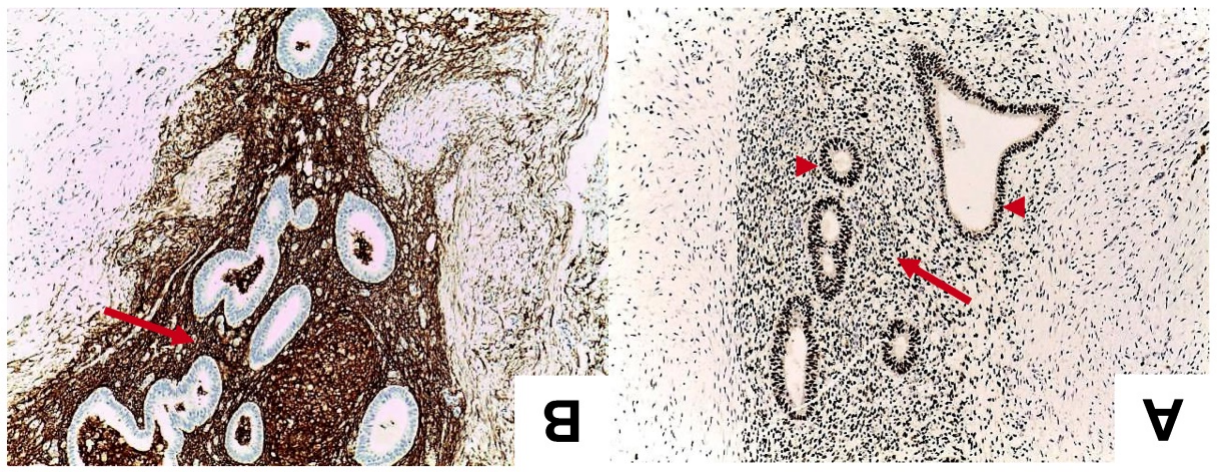
Abdominal wall endometriosis. Immunohistochemistry report. A. High oestrogen receptor positivity in the epithelium of the endometrial glands (arrow heads) and in stroma (arrow). Cell nuclei are stained intensely for estrogen receptors (10x). B. CD10 positivity in the endometrial stroma (arrow) (10x).

Epithelial endometrial glands and stromal cells are positive for oestrogen receptor (ER) expression (Figure 1).

Molecular biology studies of endometriosis have shown the importance of ER as a hallmark of local changes. Endometriotic foci have oestrogen and progesterone receptors that mediate their responsiveness during the menstrual cycle. Methylation defects of genes encoding transcription factors (GATA6, steroidogenic factor-1) and ERβ cause increased production of oestrogens in the lesion, with secondary inhibition of progesterone receptor. Subsequently, retinol uptake and further metabolization are decreased, causing defects in the endometriotic tissue, with a high level of inflammation and anomalies of prostaglandin production 27. Moreover, Gou Y*et al.* showed that the activation of ERβ in stromal cells is linked to local inflammation because ER induces local CCL2 production through the NF-kB pathway, which triggers local macrophages 28. Colón-Caraballo *et al.* demonstrated that the stroma has a tendency for low expression of ERα and progesterone and high expression of ERβ in the stroma, but the ERβ: ERα ratio varies with the site of the endometriotic lesion 29.

Overall AWE is developed after surgery only by some females. The mechanisms involve local environment at the implant site including local inflammation and metalloproteinases activation due to local growth factors, estrogen stimulation through estrogen receptors and potential epigenetic changes.

## 5. Clinical onset

Specific symptoms are absent in many cases. Local pain at the caesarean scar/incision site of the abdominal wall during menstruation has been reported to be the most common complaint. Additionally, chronic pain that is unrelated to the menstrual cycle may involve not only the abdominal wall but also the pelvic and lumbar regions 30. Sometimes, the onset is an acute abdominal emergency 31. On rare occasions, a patient presents with skin changes; for instance, the patient shows ecchymosis at the level of the abdominal wall during menstruation or hyperpigmentation of a scar (with/without small local nodules) 13. A lump may be palpable at the abdominal wall, including on the post-operative scar, with a volume that may vary according to the menstrual cycle 30,32. Sometimes the lesion is not palpable, and the pain is atypical; thus, the patient is admitted in the general surgery department. Clinical diagnosis is established in 20-50% of cases, and if additional imaging methods are used, this frequency increases to 70% 11,31,32. The clinical triangle includes cyclical pain, a lump at or near the level of the scar/abdominal wall and a history of caesarean section or similar gynaecological procedures 3,11. A study by Zhang *et al.* showed that the main reason patients present with this condition is abdominal tumour identification (98.5%), followed by cyclic pain (86.9%). Almost 95% of subjects had only one lump 33. Regarding the risk factors for AWE, there is not a specific profile. A case-control study by Khan *et al.* from the Mayo Clinic, in which 2539 females who underwent surgery for endometriosis were enrolled, showed that 1.34% of the patients had AWE, most frequently (59%) of CSE type. The accuracy of the diagnosis is increased when independent risk factors, such as the presence of cyclical abdominal pain without dysmenorrhoea and a prior laparotomy, are evident 34. A study conducted by Andolf *et al.* showed that the risk for developing endometriosis after caesarean section is 1.8%. 35.

## 6. Preoperative investigations

**Figure 2:**
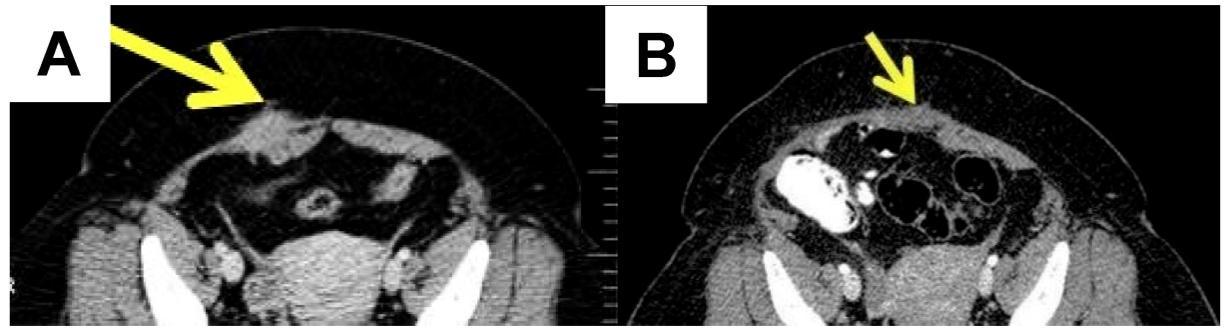
A case of a 44-year-old female diagnosed with abdominal wall endometriosis 14 years after a caesarean section. She had chronic pain unrelated to the menstrual cycle. A. Preoperative aspect: computed tomography showing a poorly defined tumour of 3.9 cm at the abdominal wall, with a heterogeneous aspect. B. Post-operative aspect by computed tomography.

If AWE is suspected, the most useful assessment tools are ultrasound, computed tomography (CT) and magnetic resonance imaging (MRI) of the abdomen, including the abdominal wall (Figure 2).

MRI is better used in cases with small lesions, while CT provides better results in cases with muscle and subcutaneous layer involvement 36. Ultrasound remains the best screening method 37. The mean diameter of the AWE was 4.7 ± 1.53 cm in one retrospective observational cohort study 38. The lesions of AWE have an isoechoic or hyperechoic pattern (46.7%), with peripheral vascularization (61.5%) on ultrasound and are homogenous and hypervascular on CT scan 39. MRI is the most commonly used method for evaluating pelvic endometriosis. It is also used for preoperative disease staging 40.

Some studies have shown the enhancement of ultrasound accuracy by elastography in the context of abdominal wall infiltration in subjects without excessive fat mass 41. Transabdominal sonoelastography appears to be particularly useful in lesions of the endometrioma type (but not in patients with a high body mass index) 42. Positron emission tomography - computed tomography (PET-CT) is less useful because of the low metabolic rate of the cells 38. Some cases of subcutaneous endometriosis have been evaluated using dermoscopy techniques 43. Additionally, for superficial lesions, ultrasound-guided fine-needle aspiration has been used depending on the anatomical profile of the lump 44,45. Fine-needle aspiration (FNA) is a simple, non-invasive, easy-to-perform procedure. For instance, in a series of 33 cases, Lopez-Soto *et al.* used FNA in 72% of cases 32. The association between cell block analysis and the cytological report has been shown, and the results of the cytological report have been improved by adding the immunohistochemistry profile based on cell block analysis 46. FNA is useful for positive diagnosis and for differential diagnosis so it may be the general case' management with a minimal risk of secondary dissemination because the procedure is minimally invasive.

Generally the screening tool remains ultrasound and as a next step MRI or CT is useful.

## 7. Pathological report

**Figure 3:**
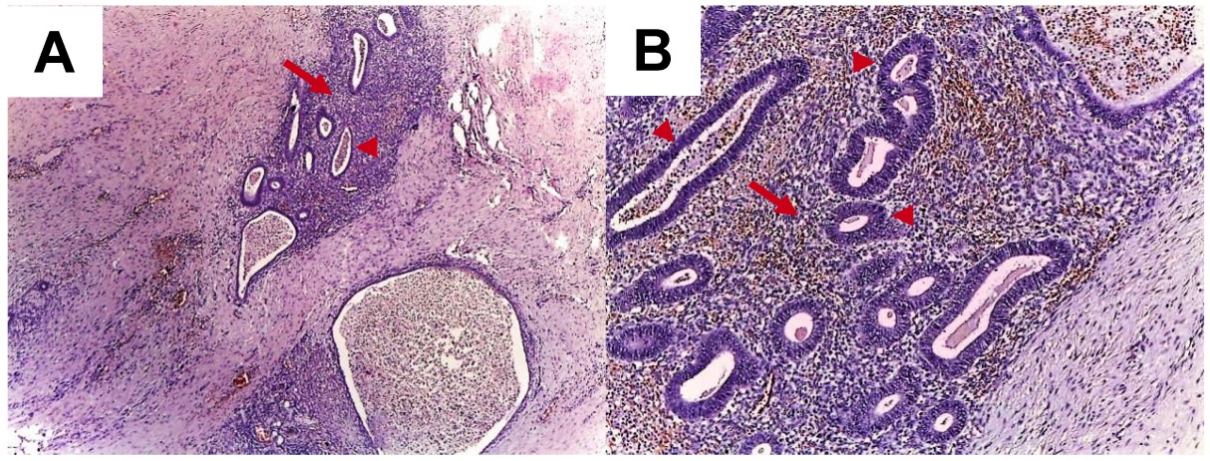
A. Abdominal wall endometriosis. Endometrial glands (arrow heads) and stroma (arrows) in the abdominal wall; HE stain, 4x (A), 10x (B).

Typically, the diagnosis is made after surgery, based on the histological report (Figure 3).

Some tumours are well defined and manifest as endometriomas 36,47,48.

In AWE, endometrial cells are implanted in the rectus abdominis muscle and into the dermis during surgery. Three AWE positions have been described in relation to the rectus abdominis: the superficial implant (above the muscle fascia), intermediate (at the level of the rectus muscle fascia), and the deep position (below the fascia) 49.

The differential diagnosis of AWE includes hernia (inguinal or incisional), abdominal wall tumours of other causes, lipomas, haematomas, granulomas, metastases from distant tumours, and desmoid tumours, among others 3,11,50,51.

## 8. Therapy

AWE requires a multidisciplinary approach. Traditionally, endometriosis is treated by hormonal therapy in addition to pain control drugs and/or surgery, depending on the purpose, namely, pain management and/or achieving fertility 1. For AWE and CSE cases, surgery is the only curative therapy, and the removal of the lump also causes chronic pain to disappear 12,48. Preoperative radioisotope injection has recently been used to clearly identify small lesions during resection but there are limited data 52. A wide incision for endometriotic nodules is recommended due to the risk of recurrence described in 5-9% of cases 16,32. Sclerotherapy with ultrasound guided ethanol injection into the lesion of scar endometriosis has been reported to be effective in isolated cases to prevent abdominal wall defects after wide excision 53. Recently, as an alternative to surgery, some authors have suggested, high-intensity focused ultrasound ablation (HIFUA), which has a recurrence rate of 3.9% 54,55. Lee JS *et al.* showed that the rate of side effects, such as blood loss and parietal defects, is lower when HIFUA is used against AWE 56. Combined oral contraceptives, progestogens and hormone suppression therapy with gonadotropin-releasing hormone (GnRH) analogues are useful for patients who refuse surgery or for post-operative management to reduce the risk of recurrence and delay new growth. Additionally, previous hormonal treatment may be an option for larger tumours and may reduce their sizes before surgery. However, the clinical improvement observed for endometriotic implants at other sites has not been observed for AWE 57. The main therapeutically approach is the surgical remove.

## 9. Malignancy risk

Endometriosis of any site has an associated malignancy risk of 1%. Eighty percent of malignancy cases are related to endometriosis located at the ovary, and 20% of these cases are related to extra-gonadal locations (including the abdominal wall) 58. Genetic anomalies, such as loss of heterozygosity or PTEN, ARID1A or p53 mutations, have been implicated 59. Local production of reactive oxygen species and prolonged oestrogen exposure may increase the risk of malignant transformation 60.

Malignant evolution is suspected in AWE cases with rapid growth of the endometriotic implant 18. In 2017, a PRISMA systematic review was published in relation to the malignancy risk of endometriosis following obstetrical surgery. This systematic review based on prior reviews and case reports included 47 cases diagnosed with AWE-related cancer between 1980 and 2016. A total of 87% of patients had a previous caesarean section, while 13% had other types of gynaecological procedures. The median period of time from surgery to cancer diagnosis was 19 years 9. Previous data suggested an interval of up to 39 years 61,62. The median survival time was 42 months, with a poor prognosis for clear cell adenocarcinoma followed by endometrioid adenocarcinoma 63,64. A prior review indicated a percentage of 44% mortality within the first few months after diagnosis 61. The treatment for endometriosis-associated malignant transformation in an abdominal surgical scar is extensive surgery and adjuvant chemotherapy and/or radiotherapy.

## 10. Conclusion

AWE represents a dynamic, yet incompletely known, multidisciplinary topic. The incidence is increasing due to the increasing number of obstetrical and gynaecological procedures. The clinical aspects range from a lump to local pain at the abdominal wall or caesarean scar. Imaging techniques like ultrasound and magnetic resonance may help but the definitive diagnosis is based on a post-operative histological report. Surgical removal of the implant currently represents the best management. The questions that still do not have a clear answer are: the true prevalence in the female population; the risk of recurrence after an initial surgical approach; the rate of malignant transformation; the underlying seeding mechanisms and pathways of cancer related. Moreover, standard protocols are needed.

## Abbreviations

AWE: abdominal wall endometriosis
ARI1A: AT-rich interactive domain-containing protein 1A
CCL2: chemokine (C-C motif) ligand 2
CYP2C19: cytochrome P450 2C19
CSE: caesarean scar endometriosis
CT: computed tomography
DNA: deoxyribonucleic acid
ER: oestrogen receptor
FNA: fine-needle aspiration
GABA6: gamma-aminobutyric acid 6
GnRH: gonadotropin-releasing hormone
HIFUA: high-intensity focused ultrasound ablation
HOXA10: homeobox protein Hox-A10
INHBA: inhibin, beta A
MRI: magnetic resonance imaging
MAPK: mitogen-activated protein kinases
NF-kB: nuclear factor kappa-light-chain-enhancer of activated B cells
PET-CT: positron emission tomography - computed tomography
PPAR-γ: peroxisome proliferator-activated receptor gamma
PI3K-Akt-mTor: phosphatidylinositol-3-kinase (PI3K)/Akt and the mammalian target of rapamycin (mTOR)
PTEN: phosphatase and tensin homolog
P53: tumor protein p53
SFRP4: secreted frizzled-related protein 4
TGFβ: transforming growth factor β
